# Damaging novel mutations in *PIGN* cause developmental epileptic-dyskinetic encephalopathy: a case report

**DOI:** 10.1186/s12887-022-03246-w

**Published:** 2022-04-25

**Authors:** Maoqiang Tian, Jing Chen, Juan Li, Hong Pan, Wenting Lei, Xiaomei Shu

**Affiliations:** 1 Department of Pediatrics, Affiliated Hospital of Zunyi Medical University, No. 143 Dalian Road, Zunyi, 563003 China; 2 Department of Cosmetic Skin Laser, Affiliated Hospital of Zunyi Medical University, Zunyi, 563003 China

**Keywords:** Multiple congenital anomalies-hypotonia-seizures syndrome, Paroxysmal nonkinesigenic dyskinesia, *PIGN* gene; seizure

## Abstract

**Background:**

Mutations in *PIGN*, resulting in a glycosylphosphatidylinositol (GPI) anchor deficiency, typically leads to multiple congenital anomalies-hypotonia-seizures syndrome. However, the link between *PIGN* and epilepsy or paroxysmal non-kinesigenic dyskinesia (PNKD) is not well-described. This study reported a patient with *PIGN* mutation leading to developmental and epileptic encephalopathy and PNKD, to expand upon the genotype–phenotype correlation of *PIGN*.

**Case presentation:**

During the first 10 days of life, a girl exhibited paroxysmal staring episodes with durations that ranged from several minutes to hours. These episodes occurred 2–5 times daily and always occurred during wakefulness. Ictal electroencephalography revealed no abnormalities, and PNKD was diagnosed. The patient also exhibited severely delayed psychomotor development and generalized seizures at the age of 4 months. Results of brain magnetic resonance imaging and metabolic screenings were normal, but trio-based whole-exome sequencing identified two novel compound heterozygous *PIGN* mutations (NM_176787; c.163C > T [p.R55 > X] and c.283C > T [p.R95W]). Flow cytometry analysis of the patient’s granulocytes revealed dramatically reduced expression of GPI-anchored proteins. This indicated that the mutations compromised GPI functions. The patient got seizure-free for 1 year, and her dyskinesia episodes reduced significantly (1–2 times/month) after treatment with levetiracetam (600 mg/day) and clonazepam (1.5 mg/day). No progress was observed with respect to psychomotor development; however, no craniofacial dysmorphic features, cleft lip/palate, brachytelephalangy with nail hypoplasia, and internal malformations have been observed until now (6 years of age).

**Conclusion:**

This is the first study to document developmental and epileptic encephalopathy with PNKD in a human with *PIGN* mutations. This report expanded our understanding of the genotype–phenotype correlation of *PIGN,* and *PIGN* may be considered a potentially relevant gene when investigating cases of epilepsy or PNKD.

## Background

The genetic etiologies of many patients with developmental and epileptic encephalopathy (DEE) are still unknown, highlighting the urgent need to identify more DEE-related genes [1]. The *PIGN* gene (OMIM* 606,097) encodes an enzyme involved in the biosynthesis of glycosylphosphatidylinositol (GPI), which anchors various proteins to the cell surface [2, 3]. Mutations in *PIGN*, resulting in a GPI anchor deficiency, typically causes multiple congenital anomalies-hypotonia-seizures syndrome (OMIM# 614,080)) and Fryns syndrome (OMIM# 229,850) [2, 3]. However, some patients with *PIGN* mutation do not fully meet the diagnostic criteria of these syndromes [4, 5]. Therefore, the phenotypic understanding of *PIGN*-related spectrum disorders needs to be improved. No cases of *PIGN*-related epilepsy with paroxysmal non-kinesigenic dyskinesia (PNKD) have been reported in humans thus far. Aiming to expand upon the genotype-phenotypic correlation of *PIGN,* we described a patient with *PIGN* mutation with DEE and PNKD without dysmorphic features.

## Case presentation

**Fig. 1:**
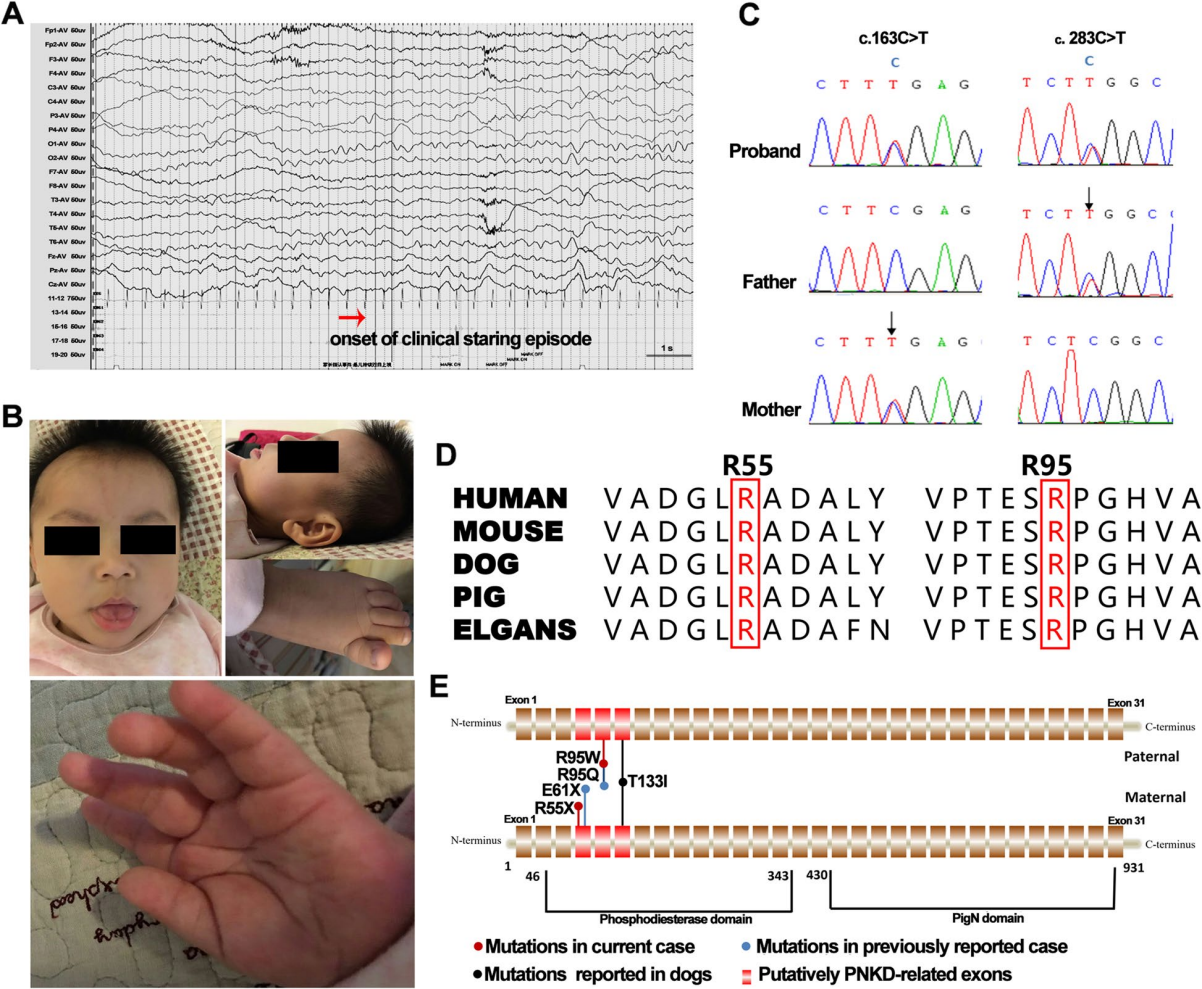
**A** Ictal electroencephalograms showing no abnormalities. **B** Photographs showing no obvious dysmorphic features at the age of 1 year. **C** The *PIGN* mutations. **D** Phylogenetic conservation of the R55 and R95 (highlighted in red). **E** Schematic of the *PIGN* gene showing the putatively PNKD-related exons. Abbreviation: PNKD, paroxysmal nonkinesigenic dyskinesia

We report the case of a girl born to healthy parents, both of non-consanguineous Asian descent. An uncomplicated pregnancy was followed by a 39-week gestation period. She was delivered by cesarean section without complications. Her birth weight was 3.5 kg (75th–90th percentile). During the first 10 days of life, the girl exhibited paroxysmal staring episodes with durations that ranged from several minutes to hours. These episodes occurred 2–5 times daily and always occurred during wakefulness. Ictal electroencephalography revealed no abnormalities (Fig. 1A). Based on the durations and frequency of her dyskinesia episodes and the apparent lack of precipitating factors, a diagnosis of PNKD was established. Developmental delay was observed at the age of 3 months, as the girl had not yet developed a social smile or control over her own head. She began experiencing generalized seizures (generalized tonic–clonic seizures) 1–2 times daily since the age of 4 months. During the initial neurodiagnostic evaluation at the age of 4 months, her weight was 6.5 kg (25th–50th percentile), height was 65.5 cm (75th–90th percentile), and head circumference was 42.0 cm (25th–50th percentile). No dysmorphic features were observed at age of 1 year (Fig. 1B), and the results of metabolic screenings were normal. Brain magnetic resonance imaging revealed no abnormalities.

Considering that the patient's early-onset epilepsy, with developmental delay, PNKD, without dysmorphic features, and hereditary causes other than chromosomal copy number abnormalities were considered, trio-based whole exome sequencing (WES) was selected for etiological exploration. The study protocol was approved by the Ethics Committee of the Affiliated Hospital of Zunyi Medical University, and the parents gave their written informed consents. The candidate variants were evaluated using the American College of Medical Genetics and Genomics guidelines [6]. The changes in protein stability were assessed using the free energy stability change (DDG, Kcal/mol) value (http://gpcr.biocomp.unibo.it/cgi/predictors/I-Mutant3.0/I-Mutant3.0.cgi). Effects of variants were predicted by Grantham scores [7] and multiple in silico tools. The conservation of mutated residues was evaluated using sequence alignment of phylogenetic species. Sanger sequencing was performed to identify potential clinically significant variants. Whole-exome sequencing identified two novel compound heterozygous *PIGN* mutations (NM_176787; c.163C > T [p.R55X] and c.283C > T [p.R95W]) (Fig. 1C): a maternally inherited nonsense mutation in exon 4 and a paternally inherited missense mutation in exon 5. Notably, the amino acid residues of the two variants are highly conserved among various species (Fig. 1D). The two variants were suggested to be damaging by at least six in silico tools (Table 1). The missense variant was predicted to have severe effects according to the Grantham scores (101), and was predicted to decrease the stability of *PIGN* with a DDG value of -0.33. Variant R55X was estimated as a pathogenic variant (PVS1 + PM2 + PP3), and variant R95W was estimated as a likely pathogenic variant (PM2 + PM3 + PP3 + PP4) based on the American College of Medical Genetics and Genomics guidelines. Flow cytometry analysis of the girl’s granulocytes revealed dramatically reduced expression of GPI-anchored proteins (34% of the expression corresponding to the control condition). This indicated that the mutations compromised GPI functions. She had no any dysmorphic features until she reached the age of 6 years. Although, she had global severe psychomotor retardation, hyperkinetic movements, non-verbal, occasionally displayed social smiles, but still couldn't control her head. Her weight was 16 kg (3rd–10th percentile), height was 112 cm (10th–25th percentile), and head circumference was 49 cm (50th–75th percentile). She was seizure-free for 1 year, and her dyskinesia episodes reduced significantly (1–2 times/month) after treatment with levetiracetam (600 mg/day) and clonazepam (1.5 mg/day).

**Table 1:** Genetic features of the individual with *PIGN* mutations

Position	cDNA change (NM_176787)	Protein change	MAF	MAF-EAS	Mutation taster	SIFT	CADD	Polyphen2	FATHMM	GERP + +	PhastCons	ClinPred
chr18:59,828,424	163C > T	R55X	4.88e-05	3.0e-04	DC (1.00)	-	D (36)	-	D (0.92)	C (2.83)	C (1.00)	-
chr18:59,824,980	283C > T	R95W	0	0	DC (1.00)	D (0)	D (34)	PD (1.00)	D (0.94)	C (4.51)	C (1.00)	P (0.99)

*Abbreviations*: *C* Conserved, *CADD* Combined annotation dependent depletion, *D* Damaging, *DC* Disease causing, *Chr* Chromosome, *MAF* Minor allele frequency from Genome Aggregation Database, *MAF-EAS* Minor allele frequency from East Asia population in Genome Aggregation Database, *NA* Not applicable, *P* Pathogenic, *PD* Probably damaging

## Discussion and conclusions

*PIGN* mapping to 18q21.33 encodes a protein with 931 amino-acids that successively contains a phosphodiesterase domain in the N-terminal and pigN domain in the C-terminal (Fig. 1E), and this protein is involved in GPI-anchor biosynthesis [3]. The GPI-anchor is a glycolipid found on various blood cells and serves to anchor proteins to the cell surface [8]. Biallelic *PIGN* variants often cause various clinical manifestations, including visceral abnormalities or dysmorphic features (> 90%), psychomotor development delay (100%), hypotonia (100%), and seizures (93%) [3, 8]. Such variants may even be lethal in the fetal or neonatal period, and can lead to multiple congenital anomalies-hypotonia-seizures syndrome and Fryns syndrome [2, 9]. However, many cases cannot be classified as either of the syndromes, as the genotype–phenotype associations of *PIGN* are not fully understood. Our patient only manifested DEE and PNKD, and no visceral abnormalities or dysmorphic features were observed until the age of 6 years. A novel term, namely developmental and epileptic-dyskinetic encephalopathy (DEDE), has been used to describe a subset of children with DEE and a hyperkinetic movement disorder including choreoathetosis, dyskinesia, dystonia, and non-epileptic myoclonus [10, 11]. DEDE is characterized by multiple types of refractory epilepsy, profound intellectual disability, and hyperkinetic movement disorder which often appears after epilepsy [10, 11]. Nevertheless, in this study, PNKD was the first presentation, which occurred independently before developmental delay and epilepsy; thus, PNKD is retained after epilepsy is controlled, indicating that *PIGN* mutations appear to be panethnic and may comprise an underappreciated cause of epilepsy and PNKD.

The *PIGN* gene is highly expressed in the brain (cerebral cortex, cerebellum, and basal ganglia) (www.proteinatlas.org/search/PIGN) and is ubiquitously expressed across the whole lifespan, with the highest expression being in the infancy stage (GD&P DataBase [gdap.org.cn]). The expressional stage of *PIGN* could explain the patient’s symptoms that gradually aggravate after birth and tend to stabilize with age. Paroxysmal dyskinesias can be attributed to abnormal basal ganglia and/or cerebellar activity [12]. Both non-acquired epilepsy and PNKD may be associated to gene mutations that affect the function of neuron membranes and lead to transient dysfunction of neuronal networks. It is speculated that these gene mutations may mainly affect the cortex-basal ganglia connection and cerebellum function, resulting in corresponding episodic presentations [12]. The high expression of *PIGN* in the cerebral cortex, cerebellum, and basal ganglia may help explain the combination of epilepsy and PNKD observed in this patient. This is the first study to document DEE with PNKD in a human with *PIGN* mutations.

The present patient carried a maternally inherited exon 4 mutation and a paternally inherited exon 5 mutation. Interestingly, a previous report described a patient with involuntary movements without dysmorphic features who harbored the biallelic *PIGN* variants involving exons 4 and 5, but the type of dyskinesia is not described in detail in this article [4]. Furthermore, the locus of the homozygous *PIGN* mutation (c.398C > T; p.T131I) previously reported in 25 affected dogs with PNKD corresponds to a locus in exon 6 in the human gene [13]. Given these findings, we can reasonably speculate that biallelic *PIGN* mutations affecting exons 4–6 may be more predisposed to cause PNKD (Fig. 1E); however, detailed molecular and phenotypic analyses of cases of *PIGN*-related disorders will be necessary to test this hypothesis.

Paroxysmal dyskinesias are characterized by recurrent episodes of involuntary dystonia, chorea, athetosis, or their combination with sudden onset and lasting a variable duration [12, 14, 15]. They are classified into three forms, namely, paroxysmal kinesigenic dyskinesia, PNKD, and exercise induced dyskinesia, based on their difference of triggers. Attacks in some patients with PNKD are triggered by unspecified factors, including emotional stress, fatigue, or consumption of alcohol or caffeine. Duration of PNKD is usually longer (up to several hours), and symptoms of onset tend to occur at a younger age compared to paroxysmal dyskinesia [14]. The *MR-1* and *KCNMA1* are reportedly related to PNKD [15–18]. The *MR-1* mutation causes PNKD without epilepsy, whereas the *KCNMA1* mutation was first reported in 2005 in a family with PNKD and epilepsy [17–19]. It was once again described in our reports of three unrelated children with PNKD and developmental delay without epilepsy [18]. Compared with *MR-1* and *KCNMA1* mutations, our patient’s *PIGN* mutations caused PNKD onset at an earlier age, including more severe DEE. Furthermore, the known pathogenetic *MR-1* and *KCNMA1* mutations are dominant, whereas our patient’s *PIGN* mutations are recessive. The clinical and genetic characteristics of patients with *PIGN* or *KCNMA1* mutations are summarized in Table 2.

**Table 2:** The clinical and genetic characteristic of patients with PNKD

Genes	*PIGN*	*KCNMA1*			
	Current case	Case 1 [18]	Case 2 [18]	Case 3^a^	Family cases [17]
Sex	Female	Male	Male	Male	10 male, 6 female
Perinatal	Unremarkable	Unremarkable	Unremarkable	Unremarkable	Unremarkable
Family	Negative	Negative	Negative	Negative	Inherited
Age of onset	10 days (PNKD)	20 days (PNKD)	7 months (PNKD)	13 months (PNKD)	6 months -15 years
Triggers	No	No	No	No	Undefined (Alcohol?)
Development milestones	Severe delay	Severe delay	Mild delay	Mild delay	NA
Diagnosis	DEE + PNKD	PNKD (till 3.5 year of age)	PNKD (till 7 year of age)	No (till 5.7 year of age)	Epilepsy and/or PNKD
Effective medicine	Levetiracetam and clonazepam	No response to oxcarbazepine, valproate and levetiracetam	Clonazepam	Clonazepam	NA
**Genetic data**					
cDNA	163C > T; 283C > T (NM_176787)	2650G > A (NM_1161352)	3158A > G (NM_1161352)	3158A > G (NM_1161352)	1301A > G (NM_1161352)
Protein	R55X; R95W	E884K	N1053S	N1053S	D434G
Inheritance manner	Autosomal recessive (inherited)	Autosomal dominant (de novo)	Autosomal dominant (de novo)	Autosomal dominant (de novo)	Autosomal dominant (inherited)

^a^This case was published in Chinese by our team

*DEE* Developmental epileptic encephalopathy, *PNKD* Paroxysmal nonkinesigenic dyskinesia, *NA* Not available

Patients with PNKD showed good response to clonazepam or diazepam [15, 18, 19]. Our patient also showed a good response to clonazepam, indicated by the reduction in the frequency of attacks from several times a day to 1–2 times per month. The patient’s epilepsy started early, but the progress of psychomotor development was not observed even the epilepsy was controlled, suggesting that *PIGN-*related DEDE was a more severe phenotype of DEE.

Our findings indicate that altered GPI anchor functions can be associated with epilepsy and PNKD, and DEDE may be one of the phenotypes of *PIGN*. This information enriches our understanding of the clinical and genetic spectrum of epilepsy and PNKD, and of the phenotypic consequences of *PIGN* mutations. We therefore conclude that *PIGN* should be considered as a candidate gene for screening in patients with early-onset epileptic encephalopathy and/or PNKD.

## Data Availability

The data that support the findings of this study will be available from supplementary materials and the corresponding author upon reasonable request.

## Abbreviations

DEDE: Developmental and epileptic-dyskinetic encephalopathy
DEE: Developmental and epileptic encephalopathy
PNKD: Paroxysmal nonkinesigenic dyskinesia
